# Supplementary material for: Spatial Transcriptomics Reveals Novel Mechanisms Involved in Perineural Invasion in Pancreatic Ductal Adenocarcinomas
Source: Cancers (Basel). 2025 Mar 1;17(5):852. doi: 10.3390/cancers17050852 (PMC11899704; doi:10.3390/cancers17050852)
Supplement: Supplementary file 1 [file cancers-17-00852-s001.zip › Figure_S3_Transcriptional_Programs.pdf]

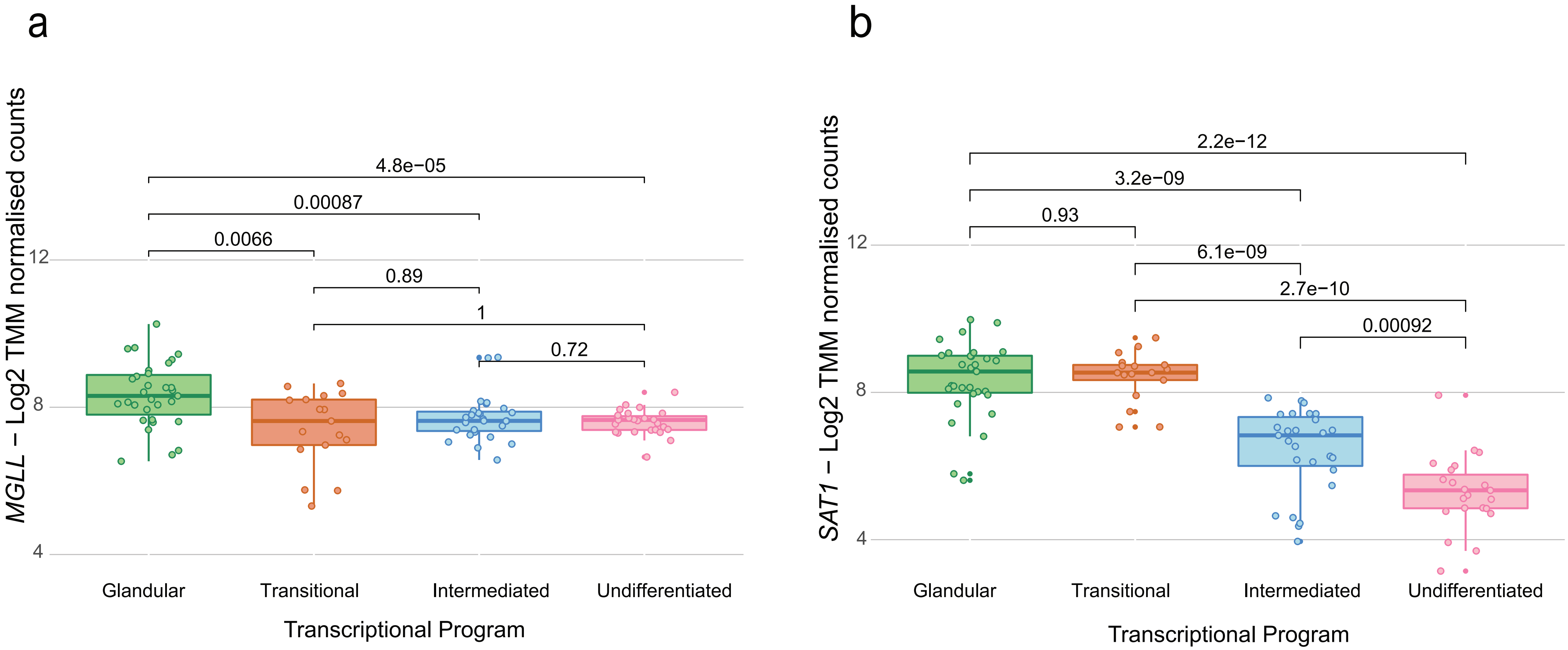

Figure S3. *MGLL* and *SAT1* expression in laser macro-dissected PDAC cancer cells RNASeq data(21). Raw counts were downloaded from Gene Expression Omnibus (GSE208732) and TMM normalised. a) *MGLL* gene expression in cancer regions enriched for each of the four transcriptional programs described by Di Chiaro et al (21). b) *SAT1* gene expression across the four transcriptional programs. *MGLL* and *SAT1* gene expression was higher in cancer cells with "*Granular"* and "*Transitional"* transcriptional programs, which were showed to be enriched in the PNI TME(21). This data support our findings that *MGLL* and *SAT1* was up-regulated in the cancer compartment of PNI compared to non-PNI foci.
