# Supplementary material for: Spatial Transcriptomics Reveals Novel Mechanisms Involved in Perineural Invasion in Pancreatic Ductal Adenocarcinomas
Source: Cancers (Basel). 2025 Mar 1;17(5):852. doi: 10.3390/cancers17050852 (PMC11899704; doi:10.3390/cancers17050852)
Supplement: Supplementary file 1 [file cancers-17-00852-s001.zip › Supplemetary_Figure_S6_Receptor_ligand_Cancer compartment_revised.pdf]

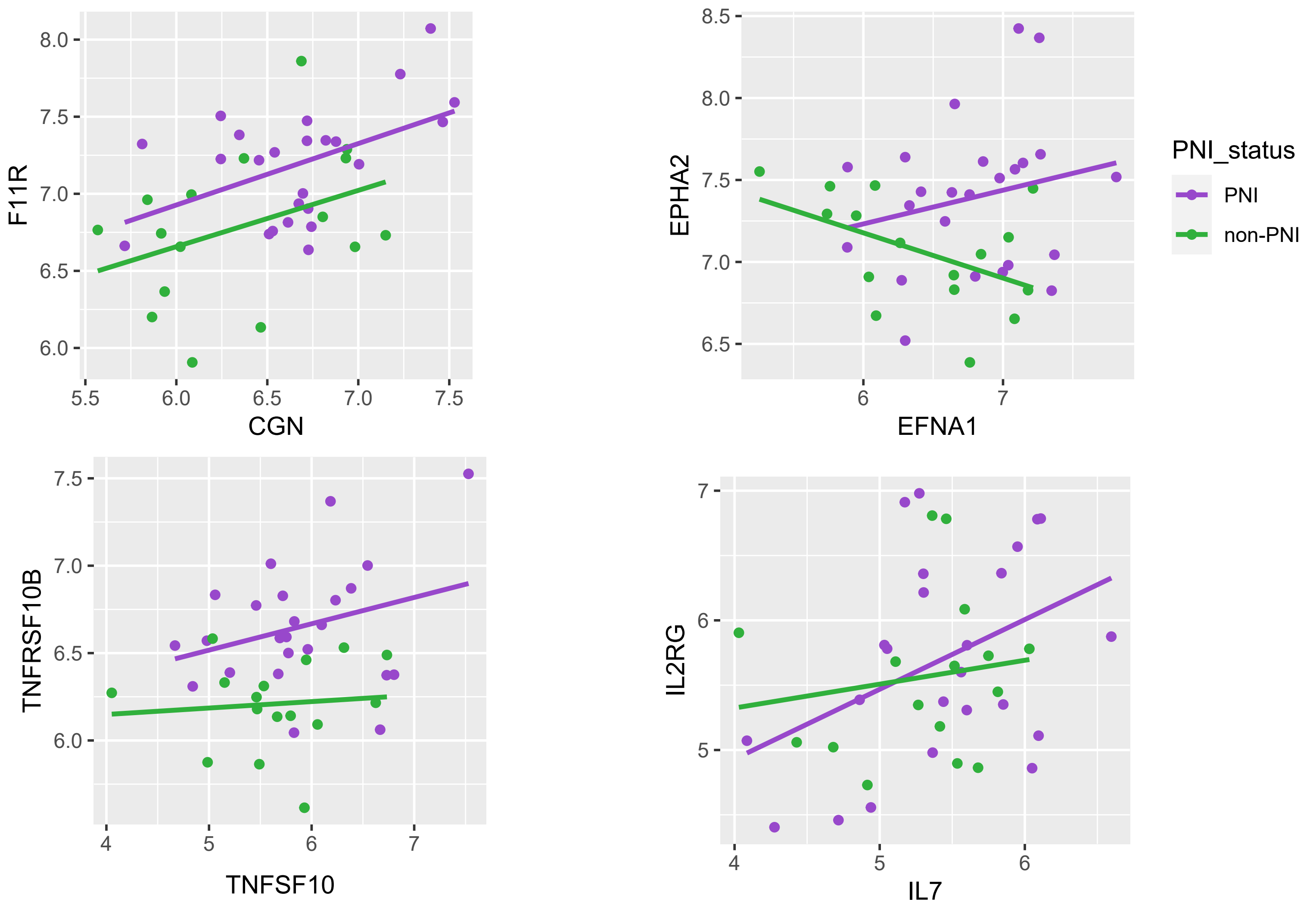

Supplementary Figure S6. Scatterplots of normalised gene expression of significant differentially expressed Ligand/Receptor in PNI and non-PNI regions were identified using BulkSignalR (adjusted p value <0.20, Supplementary Table 9) with superimposed linear model line. The trend line best represents the relationship of expression between receptor and ligand in PNI or non-PNI regions.
