# Supplementary figures and images for: Spatial Transcriptomics Reveals Novel Mechanisms Involved in Perineural Invasion in Pancreatic Ductal Adenocarcinomas
Source: Cancers (Basel). 2025 Mar 1;17(5):852. doi: 10.3390/cancers17050852 (PMC11899704; doi:10.3390/cancers17050852)

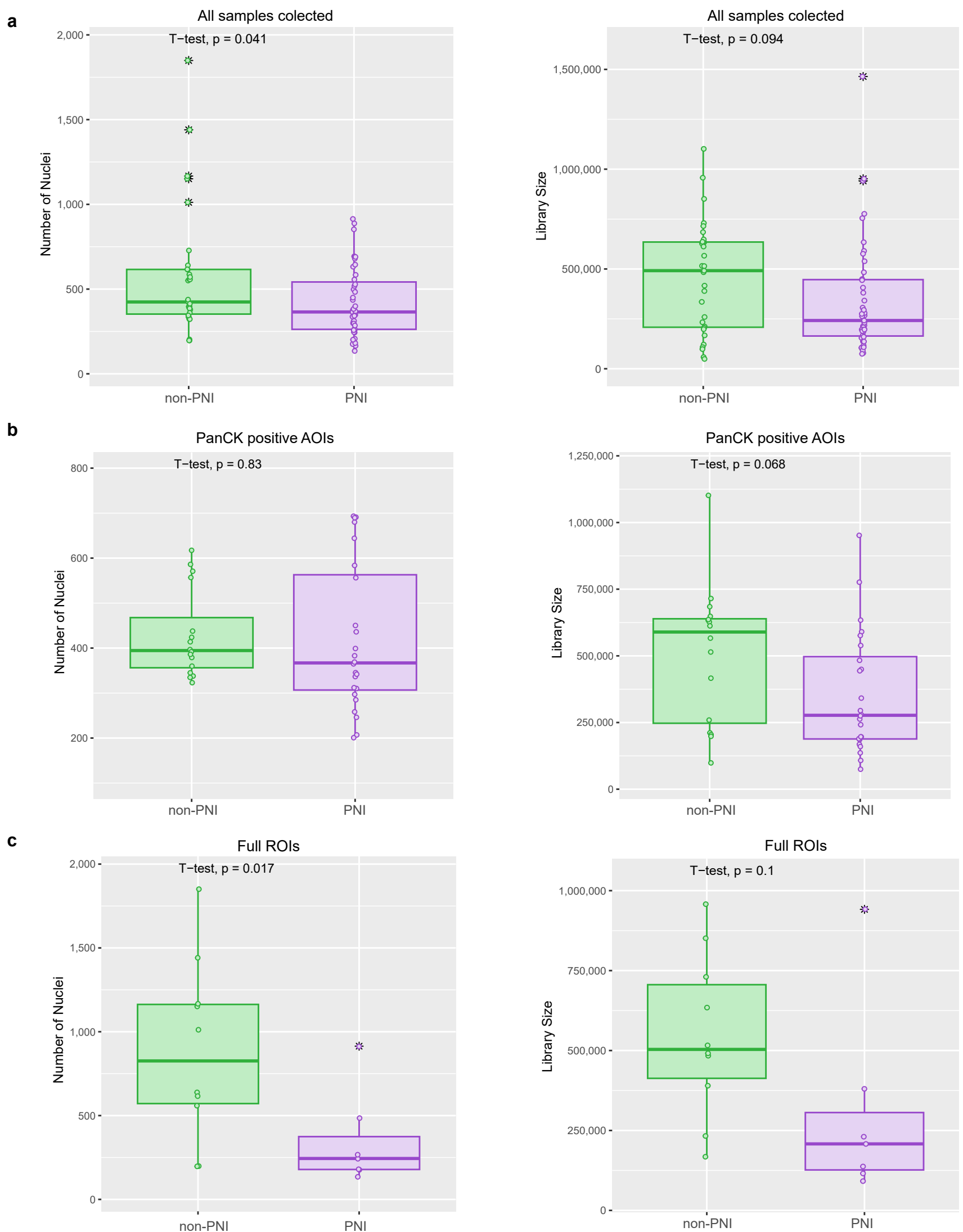

Supplement: Supplementary file 1 [file cancers-17-00852-s001.zip › Figure_S1_nuclei_library sizes.pdf]

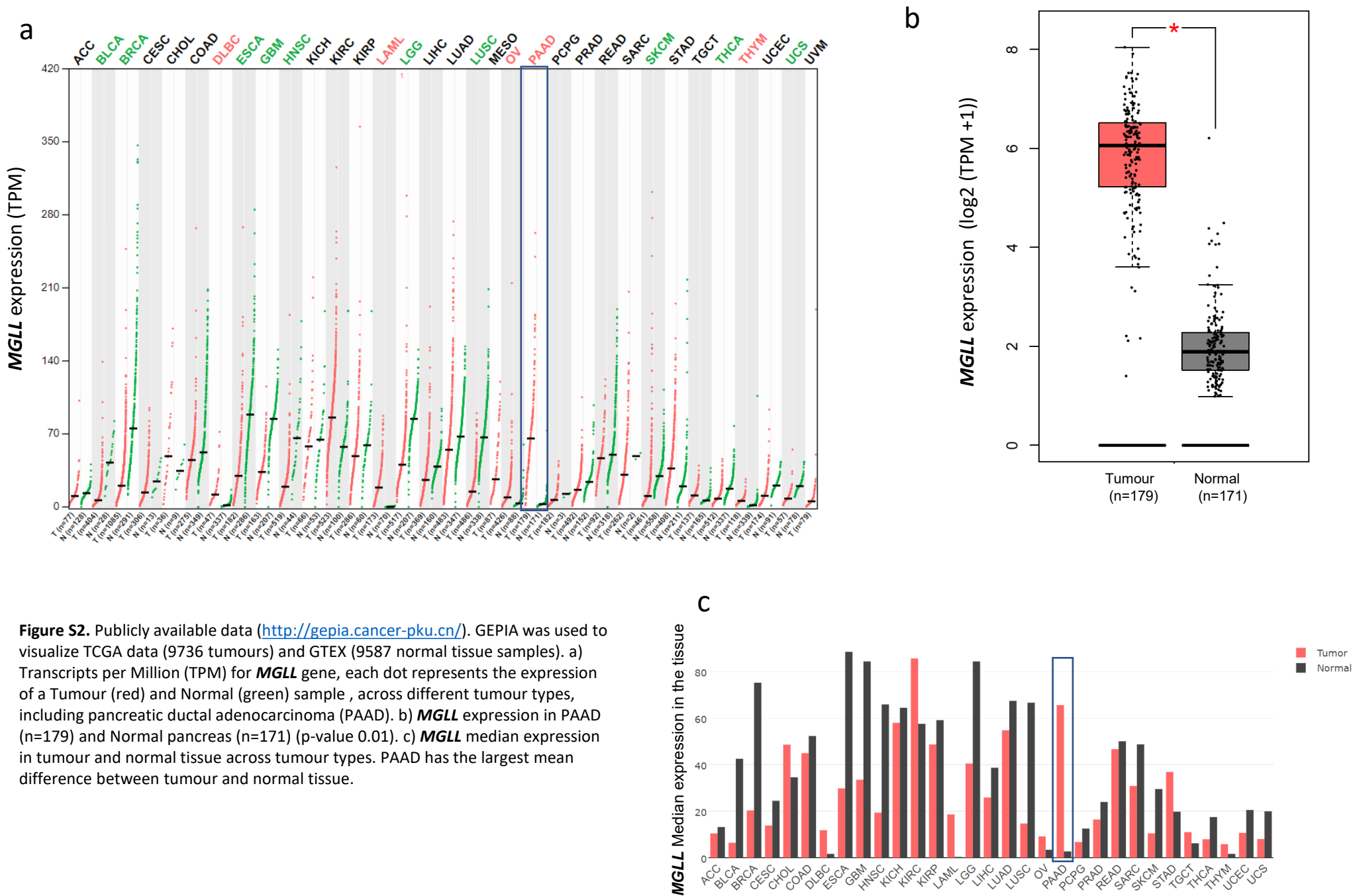

Supplement: Supplementary file 1 [file cancers-17-00852-s001.zip › Figure_S2_MGLL_expression.pdf]

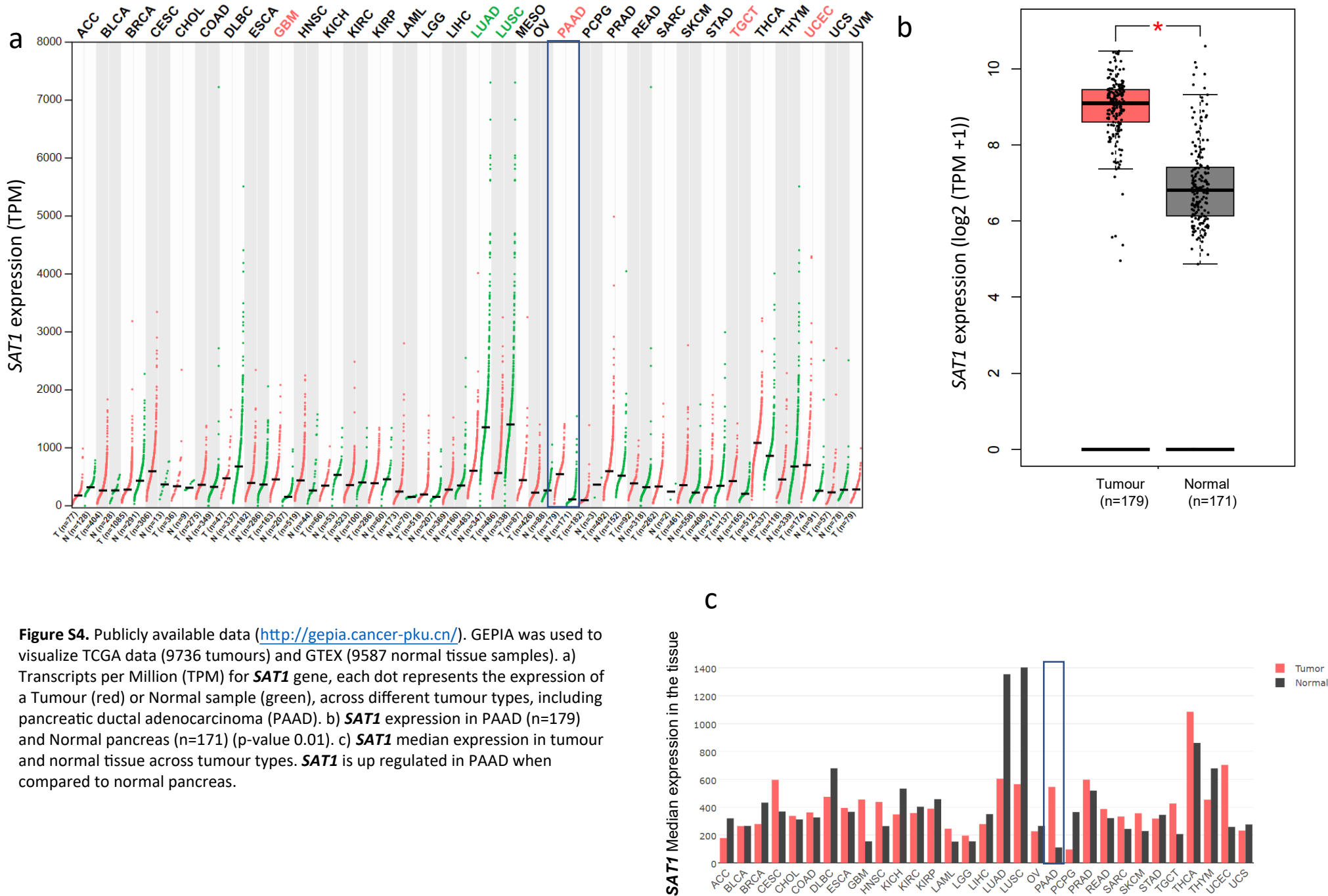

Supplement: Supplementary file 1 [file cancers-17-00852-s001.zip › Figure_S4_SAT1_expression.pdf]

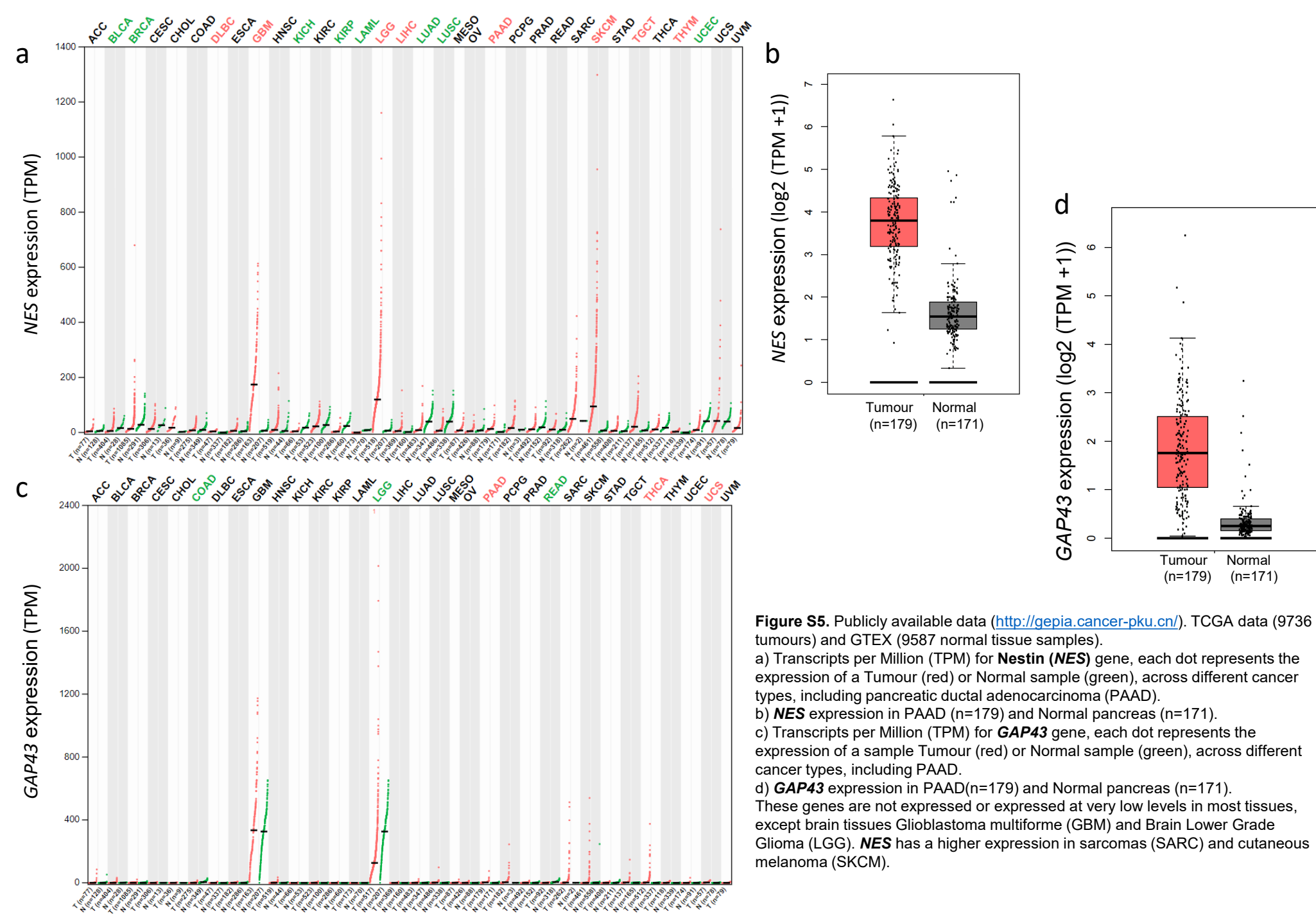

Supplement: Supplementary file 1 [file cancers-17-00852-s001.zip › Figure_S5_Nestin_GAP43.pdf]

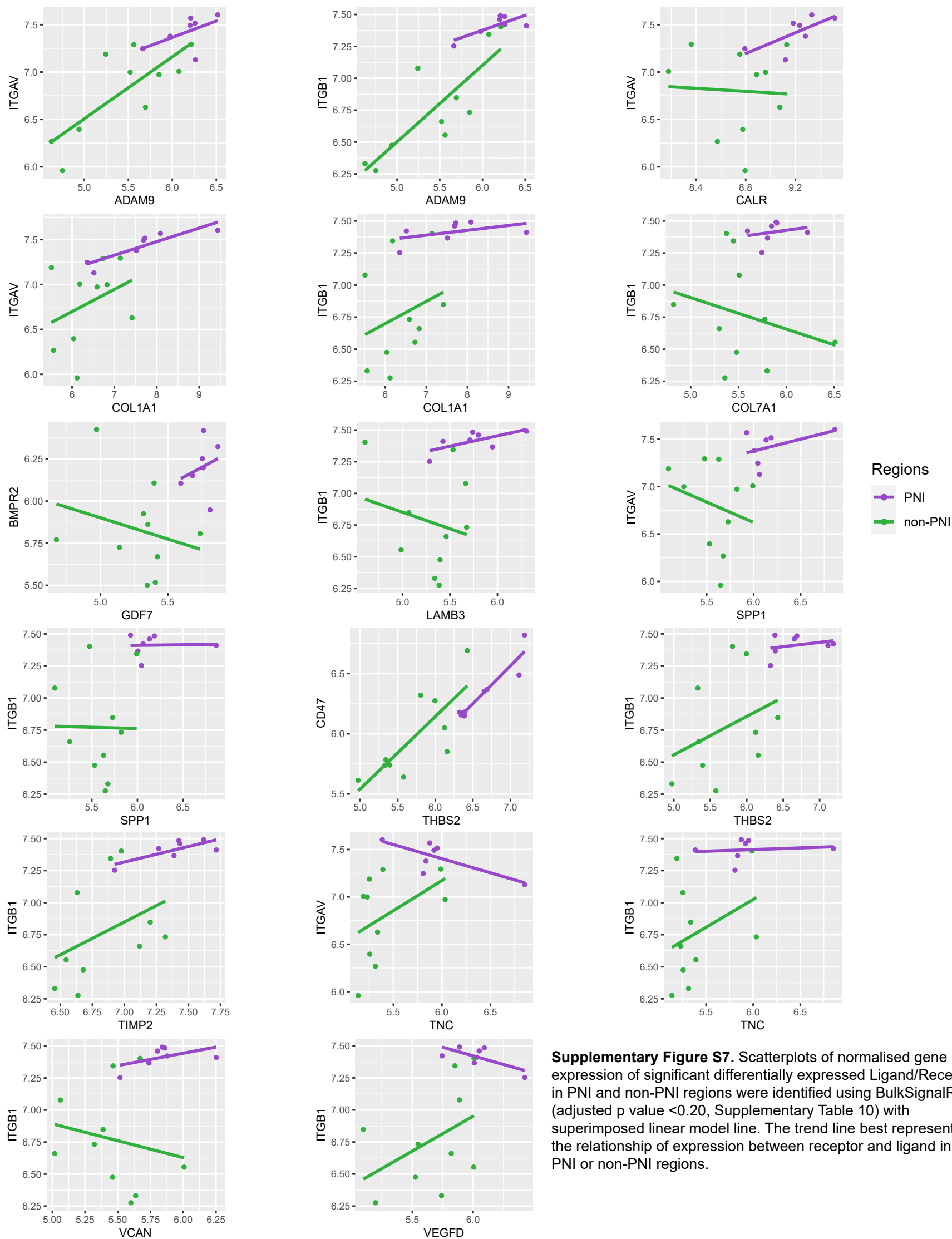

Supplement: Supplementary file 1 [file cancers-17-00852-s001.zip › Supplemetary_Figure_S7_Receptor_ligand_Nerve compartment_revised.pdf]
